# Nalfurafine is Aversive at Antinociceptive Doses in Mice

**DOI:** 10.1002/prp2.70201

**Published:** 2025-12-05

**Authors:** E. J. Kuijer, L. H. Marinelli, S. J. Bailey, D. J. Heal, S. Smith, S. Wonnacott, C. P. Bailey

**Affiliations:** ^1^ Department of Life Sciences University of Bath Bath UK; ^2^ DevelRx Ltd, BioCity Nottingham UK

**Keywords:** analgesia, antinociception, aversion, G protein bias, kappa opioid receptors, nalfurafine

## Abstract

Nalfurafine is the only clinically approved kappa opioid receptor (KOPr) agonist that can cross the blood–brain barrier and exert CNS effects. Because its clinical use is not associated with dysphoria, it is widely believed to have an atypical pharmacological profile. Nalfurafine's atypical properties are proposed to result from its G‐protein‐biased KOPr agonist property, leading to the widespread use of nalfurafine as a nonaversive KOPr agonist in preclinical research. The validity of nonaversive claims for nalfurafine was investigated in mice by comparing its antinociceptive and aversive effects with those of the typical, nonbiased KOPr agonist U50,488 in tail withdrawal and conditioned place aversion (CPA) tests. Dose responses for tail withdrawal with nalfurafine and U50,488 were determined in warm (52°C) water in adult male and female C57BL/6J mice. Doses of U50,488 produced antinociception from 5 mg/kg, and doses of nalfurafine from 0.06 mg/kg. Four‐fold lower doses of either KOPr agonist (U50,488: 1.25 mg/kg; nalfurafine: 0.015 mg/kg) were subthreshold for antinociception. No sex differences were seen. Antinociceptive effects were fully blocked by the KOPr antagonist norBNI (10 mg/kg). Antinociceptive doses of nalfurafine (0.06 mg/kg) and U50,488 (5.0 mg/kg) both induced CPA. Subantinociceptive doses of nalfurafine (0.015 mg/kg) and U50,488 (1.25 mg/kg) were nonaversive in CPA. Thus, in mice, at doses that are antinociceptive, CPA was evident for both KOPr agonists. Neither nalfurafine nor U50,488 showed a separation between their antinociceptive and aversive effects, contradicting the hypothesis that nalfurafine is a nonaversive analgesic in mice. The findings caution against assuming nalfurafine is a nonaversive KOPr agonist for use in preclinical research.

**Abbreviations**
CPA: conditioned place aversion
DI: discrimination index
GPCR: G protein‐coupled receptor
KOPr: kappa opioid receptor
MPE: maximum possible effect
norBNI: norbinaltorphimine
U50: U50,488

## 1 Introduction

Nalfurafine is a potent, brain‐penetrant kappa opioid receptor (KOPr) agonist that has garnered significant interest as the only centrally‐acting KOPr agonist to achieve clinical approval. Nalfurafine (Remitch), also known as TRK‐820 in earlier literature sources [1], is an approved drug in Japan for alleviation of uremic pruritus caused by kidney or peritoneal dialysis or chronic liver diseases [2, 3, 4, 5, 6, 7, 8] for review see [9]. Notably, whilst clinical use of the KOPr agonist class has been stalled by the emergence of intolerable psychiatric adverse events such as dysphoria and psychotomimesis [10, 11, 12], these side effects were not observed in clinical trials of nalfurafine for pruritus [2, 3, 4, 5, 6, 7, 8, 13], for review see [14]. This has led to the widespread assumption that nalfurafine is an anomaly among KOPr agonists in being able to exert therapeutic effects without inducing dysphoria.

The prevailing hypothesis to explain nalfurafine's apparent nondysphoric profile is because of its G‐protein/arrestin signaling bias at the KOPr. Evidence has emerged from basic research suggesting that the G‐protein‐coupled pathway mediates the desired analgesic and anti‐pruritic effects of KOPr agonists [15] while the downstream β‐arrestin2 signaling pathway mediates their aversive and dysphoric effects [15, 16, 17]. However, evidence for nalfurafine's profile as a biased KOPr agonist has been hotly debated; nalfurafine has been reported to be G‐protein‐biased [18, 19, 20, 21], neutral [22], and even β‐arrestin2‐biased [23, 24]. The lack of dysphoria seen in the clinical use of nalfurafine as an anti‐pruritic has been extrapolated to other indications, and then used to argue that nalfurafine's putative G‐protein signaling bias explains its general nondysphoric action. This has led to the widespread use of nalfurafine as a nondysphoric, nonpsychotomimetic, CNS‐active, KOPr agonist tool in preclinical research.

In this study we tested the hypothesis that nalfurafine is relatively more potent at inducing antinociception than at inducing aversion (i.e., that it is ‘functionally biased’). We have evaluated the relationship between nalfurafine's antinociceptive effects in the warm water tail withdrawal test and its dysphoric effects in conditioned place aversion (CPA) in mice. In these experiments, the antinociceptive and aversive effects of nalfurafine have been compared against the conventional KOPr agonist, U50,488, which has well‐established antinociceptive and aversive effects [19, 25, 26]. If the hypothesis is true that nalfurafine produces functionally selective effects, we would expect to see a separation in doses that produce antinociception versus those that produce aversion, which would contrast with the expected lack of separation of doses for the typical KOPr agonist U50,488. On the other hand, if nalfurafine does not show any dose separation, and in this regard mimics U50,488, we would conclude that nalfurafine is not an atypical agonist, which would constrain its credentials for preclinical use.

## 2 Materials and Methods

### 2.1 Animals

Adult male and female C57BL/6J mice (8 weeks of age at the start of the experiments) were initially purchased from Charles River and bred in‐house at the University of Bath, UK for more than 10 years. Separate animals were used for the tail withdrawal and CPA experiments. At weaning, mice were housed in mixed litter groups of five (tail withdrawal experiments) or four (CPA experiments) in cages (30 × 16 × 14 cm) with wood shavings and nesting material. Mice were maintained in a behavioral holding room with controlled temperature (21°C ± 1°C), humidity (40%–50%), a 12:12 h light–dark cycle (lights on: 0700–1900) and food and water given ad libitum. Animals were handled daily for 3 days and weighed on the last day prior to the start of experiments. Experiments were performed between 10:00 and 16:00 h and mice were habituated to the behavioral room for 30 min in the tail withdrawal, or 15 min for CPA experiments. All experiments were performed in accordance with the UK Animals (Scientific Procedures) Act of 1986 and approved by a local ethical review panel.

### 2.2 Drugs

(±)‐U50,488 hydrochloride [trans‐3,4‐dichloro‐N‐(2‐(1‐pyrrolidinyl)cyclohexyl)‐benzeneacetamide], further referred to as U50,488, was obtained from Tocris (Bristol, UK) and dissolved to a final concentration of 0.125, 0.5, or 2 mg/mL. Nalfurafine (Adooq Bioscience, Irvine, CA, USA) was dissolved to a final concentration of 0.0015, 0.006, or 0.024 mg/mL. norBNI (Tocris, Bristol, UK) was dissolved to a final concentration of 1 mg/mL. All drugs were dissolved in sterile isotonic sodium chloride solution (0.9% w/v, Hameln pharmaceuticals, Gloucester, UK) and filter sterilized using 0.45 μm syringe filters (Millipore, Feltham, UK). All drugs were administered intraperitoneally (i.p.) at a dose volume of 10 mL/kg.

### 2.3 Warm Water Tail Withdrawal

KOPr‐induced spinal antinociception was assessed using the warm water tail withdrawal test [18]. Animals were habituated for 30 min before the onset of the experiment and handled by tail. The latency (s) to withdraw the tail from 52°C water was measured. A cut‐off time of 15 s was used to avoid tail tissue damage. For each mouse, once a day, the baseline latency was obtained and immediately followed by administration of either nalfurafine (0.015–0.24 mg/kg), U50,488 (1.25–20 mg/kg), or saline (10 mL/kg) with injection sites alternating between left and right sides over odd and even days. Animals were retested for tail withdrawal latency 30 min later. Injections were randomized using Latin Square randomization in which the order in which drugs were tested was completely counterbalanced per animal and cage. Animals were split into two cohorts. The first cohort received five daily injections over 5 days (10 males and 10 females, nalfurafine: 0.015, 0.06, 0.24 mg/kg, U50,488: 20 mg/kg, or saline); the second cohort received four daily injections over 4 days (10 males and 10 females, nalfurafine: 0.06 mg/kg, U50,488: 1.25, 5 mg/kg, or saline). Drug administrations were spaced at least 24 h apart to ensure a complete washout of U50,488 and nalfurafine [27, 28, 29]. To investigate whether the antinociceptive effects of nalfurafine and U50,488 were through KOPr activation, a separate cohort underwent the tail withdrawal assay as described previously but here received norBNI (10 mg/kg) or saline (10 mL/kg) 24 h before nalfurafine (0.24 mg/kg) or saline. Seventy‐two hours after norBNI administration animals were administered U50,488 (20 mg/kg) or saline. The experimenter was masked to treatments throughout.

### 2.4 KOPr Agonist‐Induced Conditioned Place Aversion (CPA)

Place conditioning was performed as previously described [30]. Briefly, the CPA apparatus consisted of two distinct compartments (15 × 15 cm) with either horizontally black‐and‐white striped walls and a mesh floor, or gray walls with a different mesh floor connected by a removable door (Ugo Basile, Gemonio, Italy). All experiments were carried out in sound‐attenuating boxes under dim lighting. Animals were habituated to the room for 15 min before the onset of the experiment and handled by tube.

After 3 days of handling, mice were introduced to the CPA apparatus and had access to both chambers for 15 min on two consecutive days (habituation). Time spent in both chambers was recorded using tracking software EthoVision XT version 17.0 (Tracksys). Animals were pseudo‐randomized into the treatment groups based on similar preference in time spent in each compartment during habituation between cohorts. Doses of KOPr agonists were selected based on the results of the tail withdrawal experiments. Mice were conditioned to receive U50,488 (1.25 or 5 mg/kg, i.p.) or nalfurafine (0.015 or 0.06 mg/kg, i.p.) in one of the two chambers, counterbalanced and pseudorandomised over preference during the pre‐test sessions. The experimenter was masked to treatments throughout. Mice were conditioned over four, once‐daily, conditioning sessions and randomly assigned to receive drugs on either the odd (Days 1 and 3) or even (Days 2 and 4) conditioning days. On the drug‐paired days, mice were pre‐treated with either U50,488 (1.25 or 5 mg/kg, i.p.) or nalfurafine (0.015 or 0.06 mg/kg, i.p.) immediately before 40 min of confinement to their KOPr agonist‐paired compartment (door shut). On the saline‐paired days, animals were pre‐treated with 0.9% w/v saline (10 mL/kg, i.p.) prior to 40 min of confinement to the nondrug‐paired compartment (door shut). Following 4 days of conditioning, KOPr agonist‐induced aversion was tested by reintroducing the mice to the apparatus for 15 min (doors open) and assessing time spent in both compartments. Mice were subsequently killed by cervical dislocation.

### 2.5 Statistical Analysis

Data were analyzed using Microsoft Excel (2019) and GraphPad Prism 9.4.1 (La Jolla, CA). All *p*‐values were taken two‐tailed and significant at *p* < 0.05. KOPr‐agonist‐induced changes in latency to withdraw the tail were converted to % max possible effect: [(test latency—baseline latency)/(cut‐off (15 s) – baseline latency)] × 100% and plotted against log dose values. Dose–response curves were generated using least‐squares linear regression analysis followed by calculation of 95% confidence intervals (95% CI) and subsequent derivation of SEMs [31] after outliers, identified by Grubbs test, were removed (*n* = 1). ED_30_ values and 95% confidence intervals were derived from generated dose–response curves. ED_30_ values, rather than ED_50_, were used as the highest experimental values for both agonists were at ~50%–70% Maximum Possible Effect (MPE); thus ED_30_ values allow for comparison between KOPr agonists. Antinociception by 5 mg/kg U50,488 was compared to other doses of nalfurafine using unpaired *t*‐tests. Subthreshold doses were determined as statistically indistinguishable from 0 by one‐sample *t*‐test. Sex differences in KOPr agonist‐induced dose–response curves were investigated by comparing extra sum‐of‐squares *F* test. Effects of norBNI on KOPr agonist‐induced thermal antinociception were compared using one‐way ANOVA test with Tukey's multiple comparisons.

For the place conditioning experiments, exploration time at test was measured automatically using tracking software and the preference score was calculated as discrimination index (DI). DI = (time spent in paired compartment) – (time spent in unpaired compartment)/(total time in the apparatus) where a preference score of 1 denotes all time spent in the paired compartment (perfect preference), and a score of −1 in the unpaired compartment (perfect aversion). Preference scores were tested for normal distribution with Shapiro–Wilk test and preference during CPA test (post‐drug) was compared to behavior during habituation (pre‐drug) using a paired *t*‐test. Comparisons between drugs (post‐drug) were made using unpaired *t*‐test. To evaluate the effects of sex and drug on CPA two‐way ANOVA was used. Locomotion scores were tested for normal distribution with Shapiro–Wilk test and comparisons between KOPr agonists and saline were made with one‐way ANOVA test with Tukey's multiple comparisons. Acute KOPr agonist‐induced effects on locomotion were only compared on the first day of conditioning.

### 2.6 Nomenclature of Targets and Ligands

Key protein targets and ligands in this article are hyperlinked to corresponding entries in http://www.guidetopharmacology.org, the common portal for data from the IUPHAR/BPS Guide to PHARMACOLOGY [32], and are permanently archived in the Concise Guide to PHARMACOLOGY 2019/20 [33].

## 3 Results

### 3.1 Establishing Antinociceptive and Sub‐Threshold Antinociceptive Doses of Nalfurafine and U50,488 in the Warm Water Tail Withdrawal Assay

The warm water tail withdrawal assay was used to define antinociceptive and subthreshold antinociceptive doses of nalfurafine and U50,488. Dose ranges were chosen based on previous studies in mice indicating a U50,488‐induced antinociceptive dose of 5 mg/kg [19] and ≥ 15 mg/kg [18]. Nalfurafine has previously been shown to produce thermal antinociception in doses of ≥ 0.015 mg/kg [19] or ≥ 0.05 mg/kg [18].

**FIGURE 1:**
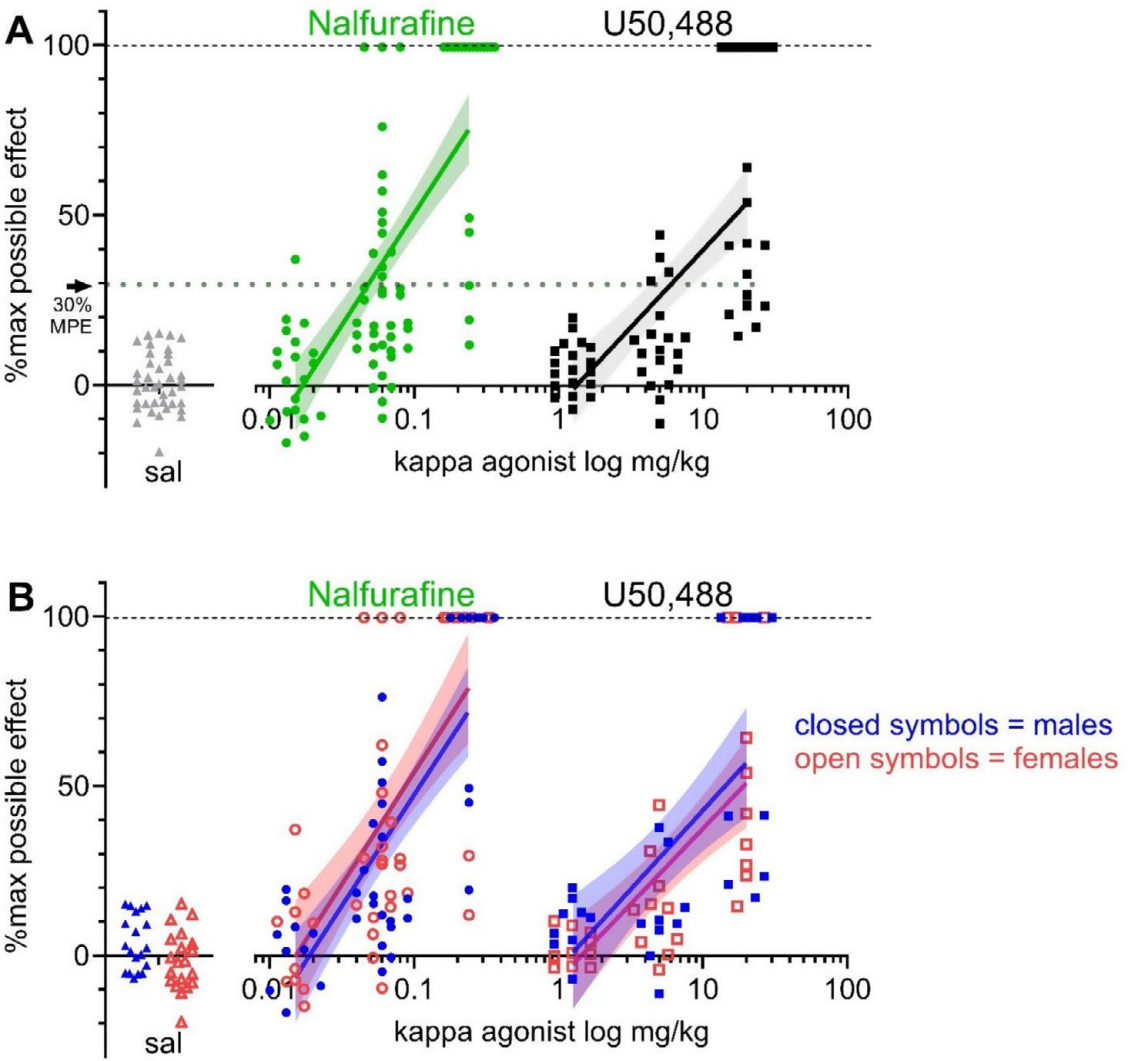
Dose–response relationships of KOPr agonist‐induced thermal antinociception in adult C57BL/6 mice. The latency to withdraw the tail from warm water is expressed as the %maximum possible effect (MPE) for male and female mice given nalfurafine (0.015–0.24 mg/kg) or U50,488 (1.25–20 mg/kg), (A) shows dose‐dependency of latency to withdraw the tail for nalfurafine (green) and U50,488 (gray) with equieffective antinociception at ED_30_ (30% MPE) indicated by the dotted line. (B) shows the same data split per sex (red, females; blue males). Data points represent individual measurements (*n* = 20–40 per treatment group) and dose–response relationships shown as a fitted linear regression (red, female line of best fit; blue male line of best fit) with 95% confidence interval (shading) according to [31].

The ED_30_ of nalfurafine was 0.048 mg/kg [95% CI: 0.038–0.059 mg/kg] and for U50,488 was 5.82 mg/kg [95% CI: 4.30–8.47 mg/kg] (Figure 1). A dose of 0.06 mg/kg nalfurafine increased the latency to withdraw the tail by 36.3% [95% CI: 30.2%–42.4%]. This mean latency was not significantly different from the latency produced by 5 mg/kg U50,488 which increased the latency by 26.5% [95% CI: 20.2%–32.8%] as compared by unpaired t‐test (*p* = 0.055) (Figure 1A). The 26.5% increase in latency by 5 mg/kg U50,488 was significantly different from the latency induced by nalfurafine at a higher dose of 0.015 mg/kg (mean: 75.8% [95% CI: 65.6%–86.0%], *p* < 0.0001) and by U50,488 at a higher dose of 20 mg/kg (mean: 54.2% [95% CI: 44.0%–64.4%], *p* < 0.0001). The lowest dose of both KOPr agonists produced no antinociception and were both statistically the same as zero, and thus subthreshold: 1.25 mg/kg U50,488 decreased the latency by −1.0% [95% CI: −11.1–9.1] (*p* = 0.85), 0.015 mg/kg nalfurafine reduced the latency by −3.3% [95% CI: −13.6–7.0] (*p* = 0.54). The slopes of the nalfurafine and U50,488 lines of best fit were not significantly different from each other (best fit [95% confidence intervals]: nalfurafine 65.9 [51.8–79.9]; U50,488 45.7 [32.7–58.7]). There were no sex differences in KOPr‐induced antinociception. Comparing linear regressions between sexes, no sex differences in %MPE induced by nalfurafine (*p* = 0.56) nor U50,488 (*p* = 0.75) were found (Figure 1B). Similarly where tail withdrawal latency time, rather than %MPE, was used there were no sex differences seen in the effects of nalfurafine or U50,488 although a small, but significant, difference in baseline latency was seen (Figure S1).

### 3.2 Nalfurafine and U50‐Induced Antinociception in the Warm Water Tail Withdrawal Assay Is Through Activation of KOPrs

**FIGURE 2:**
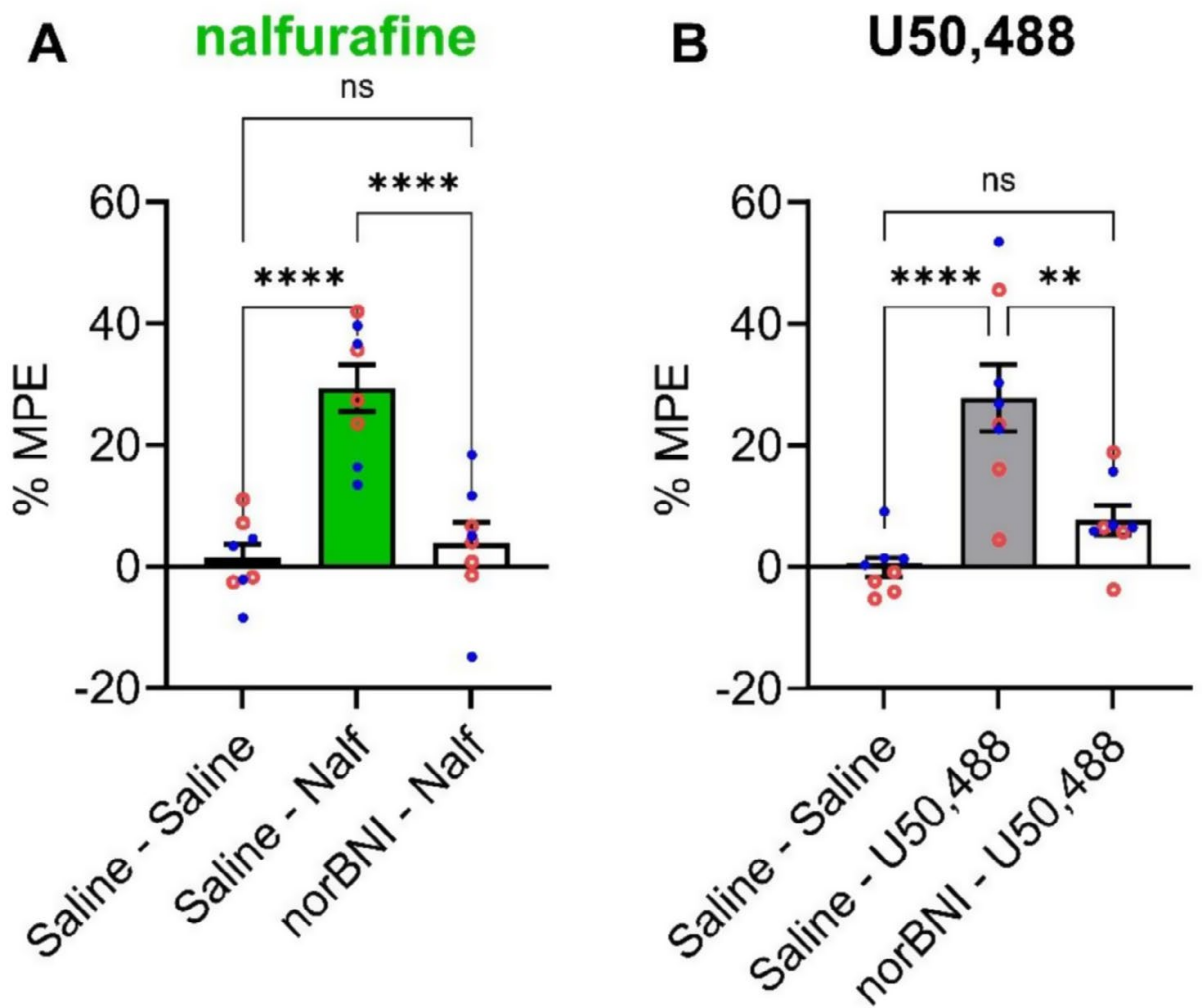
Effect of the KOPr antagonist norBNI on (A) nalfurafine and (B) U50,488‐induced thermal antinociception in adult C57BL/6 mice. The latency to withdraw the tail from warm water is expressed as the %maximum possible effect (MPE) for mice given nalfurafine (0.24 mg/kg) or U50,488 (20 mg/kg) after pre‐treatment of norBNI (10 mg/kg) (norBNI‐Nalf; norBNI‐U50,488) or saline controls (saline‐Nalf; saline‐norBNI), with saline controls (saline‐saline). Blue symbols = data from male animals, red symbols = data from female animals. **p < 0.01; ****p < 0.0001; ns = not significant, one‐way ANOVA test with Tukey's multiple comparisons (*n* = 8 per group).

To test whether the antinociceptive effects of nalfurafine and U50 were through activation of KOPrs, mice were pretreated with the long‐acting KOPr antagonist, norBNI (10 mg/kg [34]). 1–3 days after norBNI administration, both nalfurafine (0.024 mg/kg) and U50 (20 mg/kg) were ineffective in the tail withdrawal assay (Figure 2). With pooled male and female data, norBNI significantly inhibited the effect of nalfurafine (*F* (2, 21) = 22.89, *p* < 0.0001) and U50 (*F* (2, 21) = 15.01, p < 0.0001). No sex differences were seen.

### 3.3 Equieffective Antinociceptive Doses of Nalfurafine and U50,488 Produced Conditioned Placed Aversion

The doses of nalfurafine and U50,488 shown to produce supra‐threshold, equieffective antinociceptive responses in the tail withdrawal assay (0.06 and 5 mg/kg, respectively) were tested in a place conditioning paradigm, initially in male mice only. Compared with habituation to the compartments in the pre‐conditioning phase, conditioning with 5 mg/kg U50,488 produced a significant place aversion for the drug‐paired chamber, evident as a mean difference in discrimination index±SEM of −0.11 ± 0.05 (*p* = 0.049, 3A). The equieffective antinociceptive dose of 0.06 mg/kg nalfurafine also produced significant aversion (difference in discrimination index pre v post: −0.14 ± 0.02 (*p* = 0.0001, 3B)), with no significant difference in the preference score between nalfurafine‐ and U50,488‐treated animals (*p* = 0.65). An acute injection of KOPr agonist during the first conditioning session significantly affected locomotion (F (2, 21) = 16.59, *p* < 0.0001, 3C). 0.06 mg/kg nalfurafine decreased locomotion compared to saline (*p* = 0.0008) as did 5 mg/kg U50,488 (*p* = 0.0002) with no differences between KOPr agonists (*p* = 0.60, 3C).

Doses of nalfurafine and U50,488 that were subthreshold to induce antinociception in the tail withdrawal assay also did not produce significant CPA. A low, subantinociceptive dose of U50,488 (1.25 mg/kg) did not induce statistically significant CPA as compared to pre‐conditioning scores (preference score pre v post: 0.00 ± 0.07, *p* = 0.99, 3D), and neither did the low, subantinociceptive dose of nalfurafine (0.015 mg/kg) (preference score pre v post: −0.07 ± 0.07, *p* = 0.34, 3E). Furthermore, even though these doses of KOPr agonists were too low to produce antinociception or aversion, both KOPr agonists decreased locomotion on the first day of conditioning (F (2, 21) = 6.96, *p* = 0.005, 3F). 0.06 mg/kg nalfurafine decreased locomotion compared to saline (*p* = 0.01) as did 5 mg/kg U50,488 (*p* = 0.03) with no differences between KOPr agonists (*p* > 0.99, 3F).

### 3.4 No Sex Differences in Nalfurafine‐ and U50,488‐Induced Conditioned Place Aversion

**FIGURE 3:**
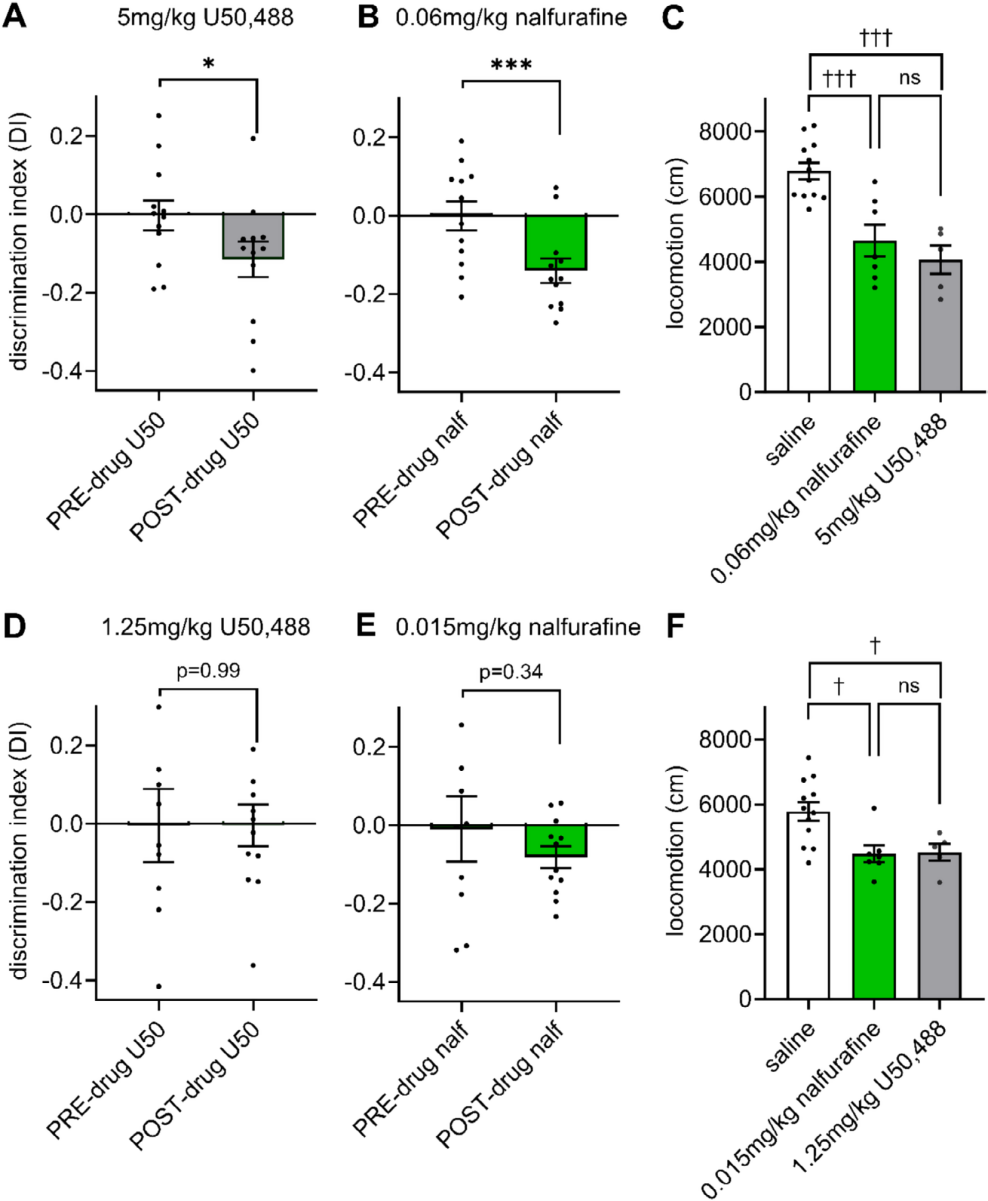
Equieffective antinociceptive doses of nalfurafine and U50,488 produce similar place‐conditioned aversion (CPA) in male adult C57BL/6 mice. Animals were place conditioned to KOPr agonists over 4 days, measured by comparing discrimination index (DI) at habituation (PRE) with DI at conditioning test (POST). (A) CPA scores after treatment with U50,488 (5 mg/kg, *n* = 12) and (B) after treatment with nalfurafine (0.06 mg/kg, *n* = 12). (C) Locomotion effects of nalfurafine (0.06 mg/kg) and U50,488 (5 mg/kg) during the first conditioning session compared to saline in cm moved during the first 15 min (*n* = 5–12/group). (D) CPA effects of a subthreshold antinociceptive dose of 1.25 mg/kg U50,488 (*n* = 12), and (E) 0.015 mg/kg nalfurafine (*n* = 12). (F) Locomotion effects of subthreshold antinociceptive and aversive KOPr agonist doses on the first conditioning day compared to saline (*n* = 5–12/group). A + B + D + E. Paired *t*‐test discrimination index between pre and post conditioning *p < 0.05; ***p < 0.001 (two‐tailed). C + F. one‐way ANOVA test with Tukey's multiple comparisons for KOPr agonist‐induced locomotion effects †*p* < 0.05; †††*p* < 0.001.

In the first set of CPA experiments (Figure 3), both U50 and nalfurafine were tested for CPA at the same time, in the same cohort of male mice. In subsequent experiments, to identify whether there were sex differences in the effects of U50 (5 mg/kg) and nalfurafine (0.06 mg/kg) CPA was investigated in male and female mice.

**FIGURE 4:**
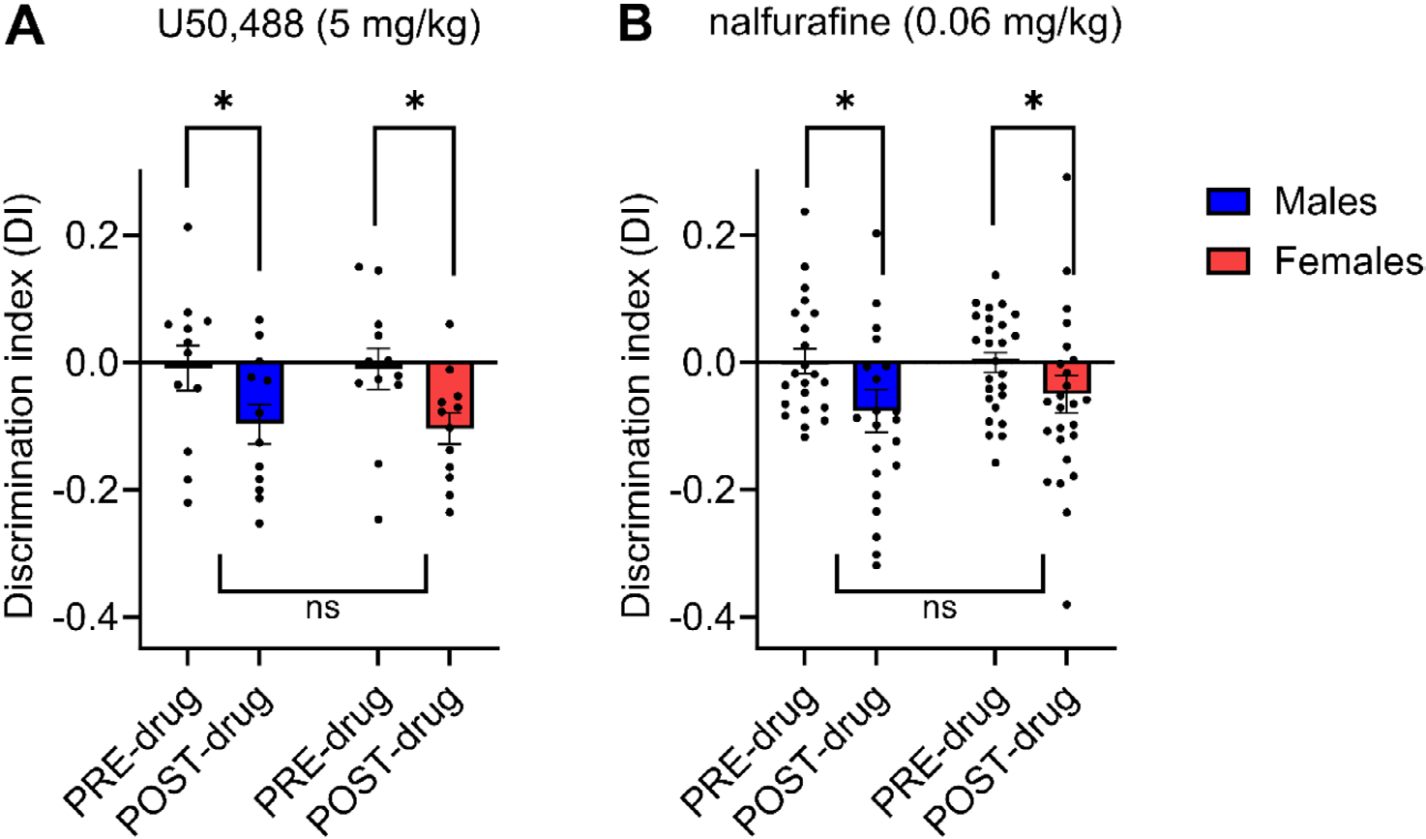
No sex differences were seen in CPA induced by equieffective antinociceptive doses of nalfurafine and U50,488 in C57BL/6 mice. Animals were place conditioned to KOPr agonists over 4 days, measured by comparing discrimination index (DI) at habituation (PRE) with DI at conditioning test (POST). (A) CPA scores after treatment with U50,488 in both male and female mice (5 mg/kg, *n* = 12) and (B) after treatment with nalfurafine in both male and female mice (0.06 mg/kg, *n* = 22–26). * = *p* < 0.05; paired *t*‐test.

As with antinociception (Figure 1) no sex‐dependent effects were observed. Figure 4 shows that U50,488 induced significant CPA in male mice (as in Figure 3A) (*p* = 0.03, Figure 4A) as well as in female mice (*p* = 0.03, Figure 4A). Similarly, nalfurafine induced CPA both in male mice (as in Figure 3B) (*p* = 0.03, Figure 4B) and in female mice (*p* = 0.04, Figure 4B). A two‐way ANOVA was performed to evaluate the effects of sex and drug on CPA (i.e., ‘post‐drug’ values). The results indicated no significant main effect for sex (F (1, 68) = 0.08, *p* = 0.78) or drug (F (1, 68) = 1.12, *p* = 0.30), and no significant interaction between sex and drug (F (1, 68) = 0.23, *p* = 0.63).

## 4 Discussion

In this study, we investigated the widely held belief that nalfurafine is a nonaversive, antinociceptive KOPr agonist, which is the basis for its extensive use in preclinical studies. Contradicting this hypothesis in mice, we demonstrate that even a moderately antinociceptive dose of nalfurafine, as determined by the tail withdrawal assay, produced conditioned place aversion (CPA) (see Table 1). Only at a subthreshold antinociceptive dose does nalfurafine fail to produce CPA; however, this low dose is still able to decrease locomotion. Based on these data, we conclude that nalfurafine and U50,488 only differ in overall potency, with nalfurafine being ~100–200× more potent than U50,488 in both antinociception and aversion in mice.

**TABLE 1:** Summary of the antinociceptive, aversive, and locomotion‐decreasing effects of nalfurafine and U50,488.

Drug (dose mg/kg i.p.)	Antinociception (tail withdrawal test)	Aversion (CPA)	Locomotion‐ decreasing effects
Subantinociceptive dose			
Nalfurafine (0.015 mg/kg)	No	No	Yes
U50,488 (1.25 mg/kg)	No	No	Yes
ED_30_ antinociceptive dose			
Nalfurafine (0.06 mg/kg)	Yes	Yes	Yes
U50,488 (5.0 mg/kg)	Yes	Yes	Yes
Supratherapeutic antinociceptive dose			
Nalfurafine (0.24 mg/kg)	Yes	N.D.	N.D.
U50,488 (20 mg/kg)	Yes	N.D.	N.D.

Abbreviations: CPA, conditioned place aversion; N.D., not determined: at this dose the mice would have been too sedated to perform in the CPA test.

The strength of our study is that we included sub‐ and supra‐threshold doses for both drugs in both assays. Our results show that the lack of separation between antinociceptive and aversive effects of nalfurafine exactly paralleled the absence of functional selectivity that is a characteristic of the conventional KOPr balanced agonist U50,488. To directly assess functional bias of GPCR agonists it is necessary to perform studies of each drug under the same experimental conditions. Few mouse studies have done this with KOPr agonists but those that have for thermal tail antinociception [18, 19, 35, 36] show that the potency ratio of nalfurafine:U50,488 is 84 to 300, matching our reported potency ratio of 121 (Table 2). Furthermore, even though other types of nociception, in particular chemically induced nociception, require a lower dose of KOPr agonist to be effective, the potency ratio between nalfurafine:U50,488 is conserved between 63 to 352 in assays across mechanical (tail/pedal pressure or pinch), thermal pedal (hot plate) and chemically induced nociception (formalin or acetic acid), in both mice and rats [22, 35, 38, 39] (Table 2), suggesting that our findings are not limited to warm water tail withdrawal. In summary, if nalfurafine is more potent in a particular antinociception assay then so is U50,488.

**TABLE 2:** Studies that have compared effective doses of nalfurafine and U50,488 in rodent behavioral assays of antinociception and aversion.

		Assay	Nalfurafine			U50,488			Strain, sex	Route of admin	PR (nalf:U50)	References
			Subthreshold dose (mg/kg)	Supra threshold dose (mg/kg)	ED50 (mg/kg)	Subthreshold dose (mg/kg)	Supra threshold dose (mg/kg)	ED50 (mg/kg)				
Mice	Thermal tail antinociception	Tail flick (52C)	0.015	0.06–0.24	0.048^a^	1.25	5–20	5.82^a^	C57BL6/J, f + m	i.p.	121^a^	Current study^a^
		Tail flick (52.5C)	0.001–0.01	0.05–0.15		1.0–7.5	15–30		C57BL/6, m	i.p.	~300	[18]
		Tail‐flick (55C)		0.015–0.06		2.5	1.25–5.0		C57BL/6J, f + m	i.p. & s.c.	n.d.	[19]
		Tail flick (56C)			0.046			8.84	SW, m	s.c.	192	[34]
		Radiant heat tail flick			0.062			5.18	ddY, m	s.c.	84	[33]
	Aversion	CPA	0.015	0.06		1.25	5		C57BL/6J, f + m	i.p.	80^b^	Current study^b^
		CPA	0.015 & 0.06	0.03			1.25–5.0		C57BL/6J, f + m	i.p. & s.c.		[19]
		CPA	0.003–0.01^c^			3^c^			C57BL6, m	i.p.		[23]
		CPA	0.00025–0.02				0.25–10		CD1, m	s.c.		[22]
	Other antinociception assays	Formalin test		0.0025–0.02	0.0058	0.25	0.5–2.5	0.58	CD‐1, m	s.c.	100	[22]
		AA writhing			0.0033			1.16	ddY, m	s.c.	352	[33]
		Tail pressure			0.0090			1.0	ddY, m	s.c.	111	[33]
		Tail pinch			0.035			11.5	ddY, m	s.c.	329	[33]
		Hot plate (51C)			0.129			8.17	ddY, m	s.c.	63	[33]
Rats	antinociception	Von Frey			0.064			11.0	Wistar, m	s.c.	160^d^	[35]
		Hot plate (52.5C)			0.2			12.7	SD, m	i.v.	63.5	[36]

*Note:* Only studies that investigated both nalfurafine and U50,488 are included. Excluded from this is [37] which compared U50,488 to nalfurafine in CPA, however only statistically quantified differences between knock‐out and wildtype animals were determined. Analysis of mean, SEM and n of CPA scores of wildtype animals revealed that a dose of 1 mg/kg U50,488 was subthreshold, whilst 2‐4 mg/kg was suprathreshold to induce CPA. Nalfurafine was subthreshold at 0.01 mg/kg, and suprathreshold at 0.02–0.04 mg/kg.

Abbreviations: 5‐HT, 5‐hydroxytryptamine; AA, acetic acid; CPA, conditioned place aversion; f, females; i.p., intraperitoneal; i.v., intravenous; m, males; n.d., not determined; PR, potency ratio; s.c., subcutaneous; SA, Swiss Albino; SD, Sprague Dawley; SLIGRL, Ser‐Leu‐Ile‐Gly‐Arg‐Leu; SW, Swiss Webster.

^a^ Comparison of U50,488 to nalfurafine is based on ED_30_ values (see text).

^b^ An estimation of potency ratio was determined by using the lowest suprathreshold dose of U50,488 (5 mg/kg) and the lowest suprathreshold dose of nalfurafine (0.06 mg/kg).

^c^ This study gave nalfurafine or U50,488 15 min before an injection of cocaine or saline before mice were put in the CPP apparatus. Only data from the control group (that received saline instead of cocaine) is included in this table.

^d^ The potency ratio stated here is as reported in the study itself.

The belief that nalfurafine is nonaversive might stem from the doses of nalfurafine used in most mouse CPA studies. All previous studies except one have used doses of nalfurafine of 0.03 mg/kg or lower, which our data indicate to be at the threshold for producing both aversion and antinociception in the CPA and tail withdrawal assays [17, 19, 22, 40, 41] (Table 2). The exception is [19], which showed that 0.03 mg/kg nalfurafine did produce significant CPA, although 0.06 mg/kg did not. These lower doses are likely based on doses used in pruritus studies, for which both nalfurafine and U50,488 are effective at lower doses [22, 42, 43], for example, [42] determined nalfurafine and U50,488 ED_50_ values of 0.0066 and 1.3 mg/kg respectively. It is important to note that the anti‐pruritic effects are largely mediated by peripheral KOPrs: difelikefalin, an anti‐pruritic KOPr agonist approved for human use, is peripherally restricted [44, 45]. Furthermore, the aversiveness of anti‐pruritic doses of nalfurafine (and U50,488) is often not determined in the same study, but the wider literature would suggest they are at, or just below, the threshold for both aversion and antinociception (Table 2). Lastly, the potency ratio between antipruritic effects of nalfurafine and U50,488 mimics that for aversion and antinociception (nalfurafine:U50 ~ *x*100–200).

Nalfurafine has been studied in other species with similar findings to those in mice. For example, using the von Frey and hot plate nociceptive tests in rats, the potency ratio of nalfurafine:U50 was 160 [38] and 64 [39]. To our knowledge no studies have directly compared nalfurafine‐ and U50‐induced antinociception with CPA in rats, although Lazenka et al. showed that an effective antinociceptive/antipruritic dose of nalfurafine also decreased performance in the intracranial self‐stimulation test [46]. Further, doses of nalfurafine that were antinociceptive in rats also caused a decrease in responding in the intravenous self‐administration test. Similarly, in nonhuman primates systemic doses from 0.0003 mg/kg are antipruritic [47, 48] but negative KOPr‐typical side‐effects including a decrease in species‐specific behavior, disrupted rest/sleep posture and increased passive visual behaviors, were seen at the same doses [48, 49]. In humans, nalfurafine's therapeutic effect might be because nalfurafine produces 250× G‐protein bias at human KOPrs compared to rodent KOPr [18]. Another possibility is that as lower doses are required for KOPr agonist‐induced antipruritic effects over antinociception, there is a narrow therapeutic window for self‐administered nalfurafine, as would be the case with other KOPr agonists.

In conclusion, we report that nalfurafine and U50,488 do not differ in their abilities to produce aversion in mice at antinociceptive doses, suggesting that nalfurafine is not functionally biased at mouse KOPrs for antinociception over aversion. This challenges the classification of nalfurafine as an atypical, nonaversive, KOPr agonist analgesic. Furthermore, these results caution against the use of nalfurafine as a centrally penetrant nonaversive KOPr agonist in preclinical research.

## Author Contributions

Conducted experiments: E.J.K., L.H.M. Drafted the manuscript: E.J.K., C.P.B. All others read, edited, and approved the final manuscript.

## Funding

This work was supported by the University of Bath and DevelRx Ltd.

## Ethics Statement

All experiments were performed in accordance with the UK Animals (Scientific Procedures) Act 1986, the European Communities Council Directive (2010/63/EU) and the University of Bath ethical review document.

## Conflicts of Interest

The authors declare no conflicts of interest.

## Supporting information


**Figure S1:** Dose–response relationships of KOPr agonist‐induced thermal antinociception in adult C57BL/6 mice (latency times).

## Data Availability

The authors declare that all the data supporting the findings of this paper is available from the corresponding author upon reasonable request.
