# Supplementary material for: Nalfurafine is Aversive at Antinociceptive Doses in Mice
Source: Pharmacol Res Perspect. 2025 Dec 5;13(6):e70201. doi: 10.1002/prp2.70201 (PMC12680513; doi:10.1002/prp2.70201)
Supplement: Supplementary file 1 — Figure S1: Dose–response relationships of KOPr agonist‐induced thermal antinociception in adult C57BL/6 mice (latency times). [file PRP2-13-e70201-s001.docx]

**Nalfurafine is aversive at antinociceptive doses in mice.**

**Kuijer EJ et al**

**Supplementary Information**

**
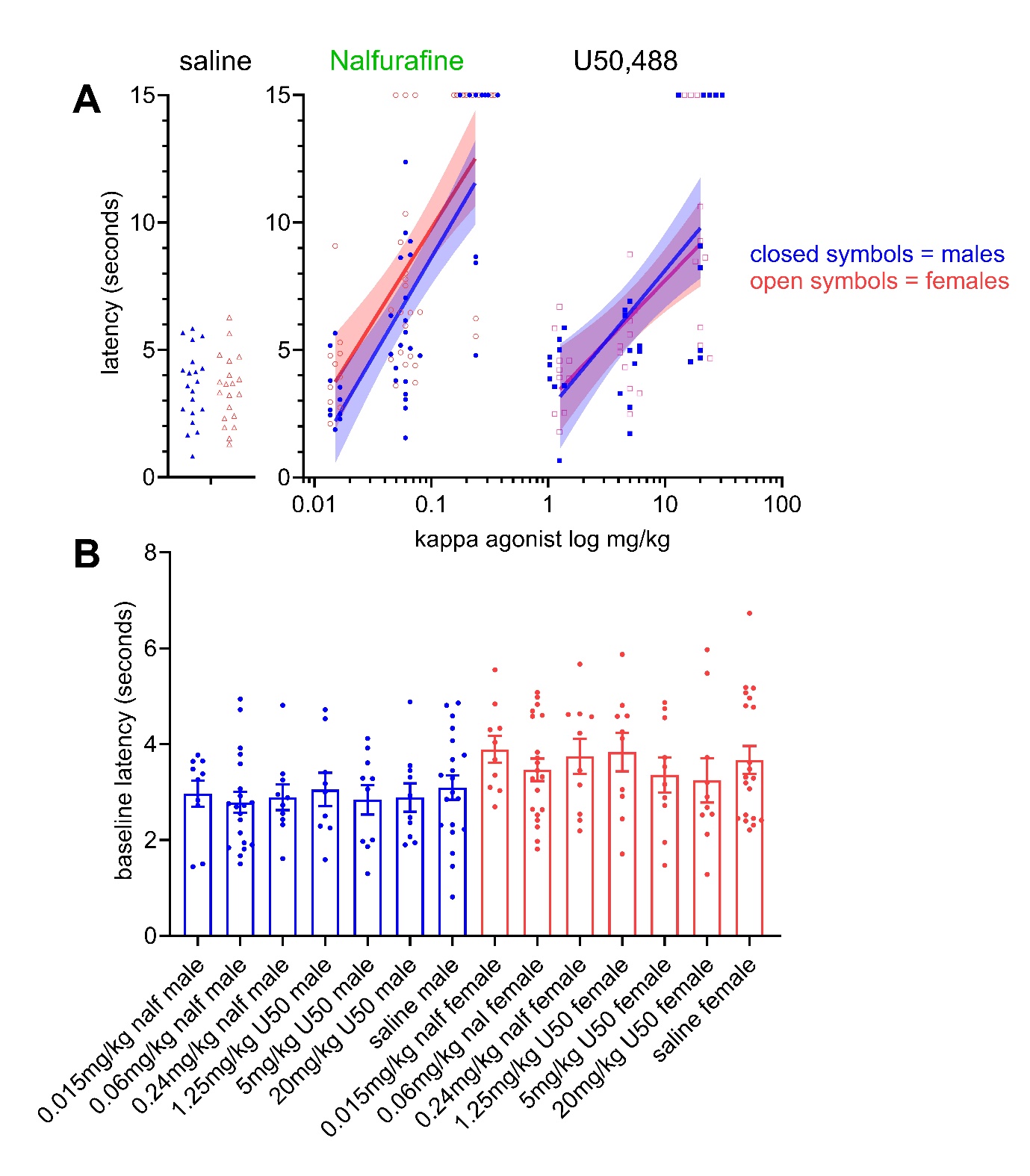
**

**Supplementary Fig. 1 Dose-response relationships of KOPr agonist-induced thermal antinociception in adult C57BL/6 mice (latency times).**

*Data as in Fig. 1 (main paper). The latency to withdraw the tail from warm water is expressed as latency time (seconds) for male and female mice given nalfurafine (0.015-0.24mg/kg) or U50,488 (1.25-20mg/kg), (A) shows dose-dependency of latency to withdraw the tail for nalfurafine and U50,488; data split by sex (red, females; blue males). Data points represent individual measurements (n=20-40 per treatment group) and dose-response relationships shown as a fitted linear regression (red, female line of best fit; blue male line of best fit) with 95% confidence interval (shading) according to (Hull et al., 2010), (B) baseline latency times for each treatment group (n=10-20 per treatment group). Pooled female versus pooled male data showed that female baseline latency time was significantly higher than male baseline latency time (3.59* *± 0.12 s v 2.93 ± 0.10 s; p<0.001 Student’s t-test). No differences were seen between individual treatment groups within each sex (female: F (6,83) = 0.46, p=0.84; male: F (6,83) = 0.21, p=0.97, one-way ANOVA).*
